# Principles of Surgical Management of Small Intestinal NET

**DOI:** 10.3390/cancers13215473

**Published:** 2021-10-30

**Authors:** Arnaud Pasquer, Thomas Walter, Laurent Milot, Valérie Hervieu, Gilles Poncet

**Affiliations:** 1 Service de Chirurgie Digestive, Hôpital Edouard Herriot, Hospices Civils de Lyon, F69437 Lyon, France; gilles.poncet@chu-lyon.fr; 2 Université de Lyon, Université Claude Bernard Lyon 1, F69100 Villeurbanne, France; thomas.walter@chu-lyon.fr (T.W.); laurent.milot@chu-lyon.fr (L.M.); valerie.hervieu@chu-lyon.fr (V.H.); 3 INSERM, UMR 1052-UMR5286, UMR 1032 Lyon Cancer Research Center, Faculté Laennec, F69437 Lyon, France; 4 Service de Gastroentérologie et d’Oncologie Digestive, Hôpital Edouard Herriot, Hospices Civils de Lyon, F69437 Lyon, France; 5 Service de Radiologie, Hôpital Edouard Herriot, Hospices Civils de Lyon, F69437 Lyon, France; 6 Service Central d’Anatomie et Cytologie Pathologiques, Hôpital Edouard Herriot, Hospices Civils de Lyon, F69437 Lyon, France

**Keywords:** small bowel neuroendocrine tumors, surgery, lymphadenectomy

## Abstract

**Simple Summary:**

Small-intestinal neuroendocrine tumors (siNETs) account for 25% of gastroenteropancreatic NETs. A total of 89% of multiple tumors are located in the ileum, often within 100 cm of the ileocecal valve. According to current guidelines, all localized siNETs should be considered for radical resection with lymphadenectomy. The preoperative workout should focus on symptoms of carcinoid syndrome (flush, diarrhea, and cardiac failure). Morphological evaluation should include a CT scan with a thin-slice arterial CT, a PET/CT with 68 Ga, and a hepatic MRI. Levels of 24 h urinary 5-hydroxyindoleacetic acid are needed. Regarding surgery, the limiting component is the number of free jejunal branches allowing a resection without risk of short small bowel syndrome. In case of emergency surgery, there is expert agreement that it is not reasonable to initiate resection of the mesenteric mass without comprehensive workup and mapping. The challenge lies in the ability to propose a resection without imposing short small bowel syndrome on the patients.

**Abstract:**

Introduction: Small-intestinal neuroendocrine tumors (siNETs) account for 25% of gastroenteropancreatic NETs. Multiple siNETs appear to develop in a limited segment of the small bowel (SB), 89% of them being located in the ileum, most often within 100 cm of the ileocecal valve (ICV). According to the European Neuroendocrine Tumor Society (ENETS) and the American Joint Committee on Cancer (AJCC), all localized siNETs should be considered for radical surgical resection with adequate lymphadenectomy irrespective of the absence of lymphadenopathy or mesenteric involvement. Surgical management of siNETs: The preoperative workout should include a precise evaluation of past medical and surgical history, focusing on the symptoms of carcinoid syndrome (flush, diarrhea, and cardiac failure). Morphological evaluation should include a CT scan including a thin-slice arterial CT, a PET/CT with 68 Ga, and a hepatic MRI in cases of suspected metastasis. Levels of 24 h urinary 5-hydroxyindoleacetic acid are needed. Regarding surgery, the limiting component is the number of free jejunal branches allowing a resection without risk of short small bowel syndrome. The laparoscopic approach has been poorly studied, and open laparotomy remains the gold standard to explore the abdominal cavity and entirely palpate the small bowel through bidigital palpation and compression. An extensive lymphadenectomy is required. A prophylactic cholecystectomy should be performed. In case of emergency surgery, current recommendations are not definitive. However, there is expert agreement that it is not reasonable to initiate resection of the mesenteric mass without comprehensive workup and mapping. Conclusion: The surgery of siNETs is in constant evolution. The challenge lies in the ability to propose a resection without imposing short small bowel syndrome on the patients. The oncological benefits supported in the literature led to recent changes in the recommendations of academic societies. The next steps remain the dissemination of reproducible quality criteria to perform these procedures.

## 1. Introduction

**Figure 1:**
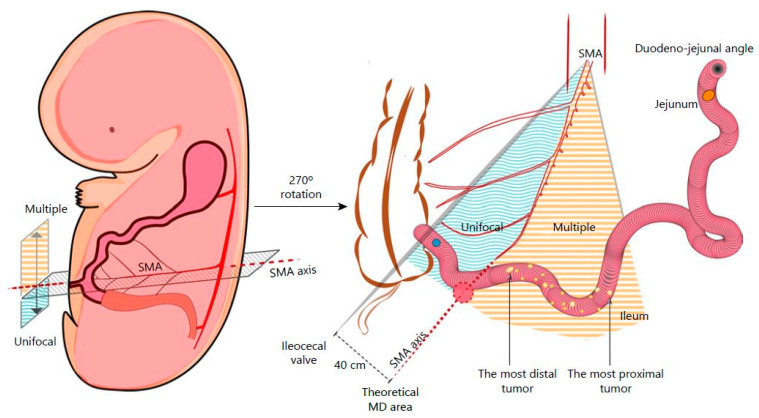
Embryological theory of single or multiple tumors. Figure issued from Kalifi, M., Walter, T., Milot, L., et al.: Unifocal versus Multiple Ileal Neuroendocrine Tumors Location: An Embryological Origin. *Neuroendocrinology* 2021; 111: 786–793. DOI: 10.1159/000511849. Reproduced with permission of S. Karger AG [10].

Small-intestinal neuroendocrine tumors (siNETs) account for 25% of gastroenteropancreatic NETs [1], but their incidence has increased by 300–500% over the past 40 years [2]. siNETs have the particularity of being multiple in 30–56% of cases and are often diagnosed at metastatic stage in 50% of cases [3,4,5,6]. There is currently no known pathogenetic mechanism underlying the development of multiple tumors, and prognosis is similar to that of unifocal siNETs [5,6]. Multiple siNETs appear to develop in a limited segment of the small bowel (SB), 89% of them being located in the ileum, most often within 100 cm of the ileocecal valve (ICV) [7]. Current Europe and Neuroendocrine Tumor Society (ENETS) recommendations propose resecting the primitive tumor(s) even when metastatic in order to prevent local morbidity (ischemia, digestive perforation, and occlusion) [8]. In small/incidentally found lesions, resection with local lymphadenectomy should be performed when pathology is unknown. In locoregionally advanced disease, radical resection with extent lymphadenectomy should be proposed due to the local evolutive risk. Resection is still debated in metastatic conditions; nevertheless, local resection at the origin of mesenteric arteries can prevent locoregional complications. Such benefits should imply discussion around surgery in metastatic context. Nodes dissections are not standardized and rely on lymph node extension. The resection of at least seven lymph nodes is correlated with an improvement in overall survival [9]. Recently, an embryological theory emerged to locate unique and multiple tumors, on the basis of the fact that multiple siNETs are mostly located on the left side of the superior mesenteric artery axis [10] (Figure 1). According to ENETS and the American Joint Committee on Cancer (AJCC), all localized siNETs require radical surgical resection with adequate lymphadenectomy irrespective of the absence of lymphadenopathy or mesenteric involvement. We here describe a standardized surgical procedure to oncologically resect those siNETs.

## 2. Scheduled Surgery

### 2.1. Preoperative Workout

**Figure 2:**
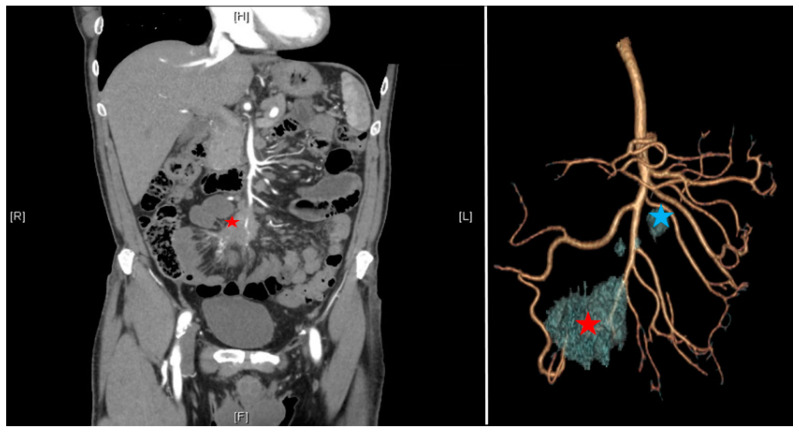
CT scan with arterial three-dimensional reconstruction; mesenteric mass (red star); proximal nodes (group 3 down from Deguelte et al. [13] in blue star).

According to the ENETS consensus guidelines [8], the preoperative workout should include a precise evaluation of past medical and surgical history, focusing on the symptoms of carcinoid syndrome (flush, diarrhea, and cardiac failure). Cardiological evaluation should be mandatory to detect tricuspid or pulmonary failure. Morphological evaluation should include a triphasic CT scan including a thin-slice arterial CT angiography (CTA) of the abdomen and pelvis allowing three-dimensional reconstruction to evaluate the vascular involvement [11,12], a PET/CT with 68 Ga (sensitivity of 90%), and a hepatic MRI in cases of suspected metastasis [8]. siNETs are sometimes difficult to see on CT scans, but mesenteric lesions appear as contrast-enhancing and surrounded by striae of desmoplastic reaction. Levels of 24 h urinary 5-hydroxyindoleacetic acid are given but not specific and used only for their prognostic utility. The interest in the three-dimensional reconstruction is multiple: it allows prediction of the extension of the lymph node involvement and anticipation of the resectability according to the Deguelte’s classification [13]. Mesenteric mass invasion is divided into four stages according to its location regarding the superior mesenteric artery (SMA): stage I: proximity to the small intestine; stage II: involvement of the distal branches of the SMA; stage III up: involvement of the trunk of the SMA with <3–4 free jejunal branches; stage III down: >3–4 free jejunal branches; stage IV: involvement of the first jejunal arteries. The limiting component is the number of free jejunal branches allowing a resection without risk of short small bowel syndrome. It is important to standardize the reconstruction technique including arterial and venous vascularization with the mesenteric mass (Figure 2). Patients who are unfit for surgery are the ones presenting peripancreatic vessel involvement (superior mesenteric vein, superior mesenteric artery, coeliac axis, and proper hepatic artery).

Patients with carcinoid syndrome received a continuous infusion of octreotide (2000 µg/day) at least 12 h before surgery, during, and at least 24 h after surgery in order to prevent perioperative carcinoid syndrome [6]. Octreotide administration aims to saturate type 2 serotonin receptors to reduce intraoperative hemodynamic risk when handling liver metastases, carcinosis lesions, or mesenteric masses. These intraoperative flushes can be fatal in the absence of saturation of these receptors.

### 2.2. Centralization of Procedures

For several years, the impact of centralization in digestive surgery on postoperative outcomes and the centralization of activity in expert centers has been debated regarding all areas of this surgical specialty, as well as in other specialties. The vast majority of these studies showed a reduction in postoperative complications and length of stay, regardless of the severity of the pathology, the type of surgery, or the patients’ comorbidities [14,15,16,17]. Reflections around centralization for siNET surgery are rising. We performed a pilot evaluation in our regional RENATEN center. It appears that lymph node resection was >12 nodes when performed in expert centers in 93% of cases versus 68%. The same observations were found regarding the rate of multiple tumors found: 46% vs. 26%. A French national study is underway to address this issue.

### 2.3. Laparotomy or Laparoscopy

The laparoscopic approach has been poorly studied. In most published studies, laparotomy is the reference approach. It allows for a much more precise evaluation of the peritoneal and mesenteric involvement if necessary; it allows for a better control of the origin of the mesenteric vessels in case of extensive nodal resection [6,18]. Similarly, systematic palpation of the small intestine is hardly feasible in laparoscopy and increases the risk of missing a small bowel lesion (up to 80% of cases after suboptimal surgery). This risk is currently being investigated by Deguelte et al. On the other hand, Kaçmaz et al. recently reported the feasibility of the laparoscopic approach in a cohort of 34 operated patients (11 open and 23 minimally invasive). A conversion to laparotomy with a 10 cm incision was necessary in 30% of the patients in the laparoscopic group because of difficulties in exposing the mesenteric root. The laparoscopic approach was safe regarding postoperative complications as well as oncological data (although in the overall cohort, five patients were classified R1 (14.7%) and two patients R2 (5.8%)). The distance between the nodal mass and the mesenteric axis did not differ between the groups. To explore the entire bowel, a 10 cm laparotomy was routinely performed to carefully palpate the small bowel. The authors concluded that the laparoscopic approach was feasible in a tertiary referral center [19]. Finally, regarding the ovarian and peritoneal metastatic implication (14% in our cohort), laparotomy appears to be the best approach in a curative intent to perform a complete cytoreductive surgery.

### 2.4. Exploration of the Abdomen

As a first step, the abdominal cavity is explored, and the full length of the small bowel is analyzed visually and through bidigital palpation and compression. Any suspected tumors are tagged with a polypropylene suture. The distance from the most proximally suspected tumor to the ileocecal valve is noted in order to predict the future length of the residual small bowel.

Then, visual searches for miliary liver metastases are systematically performed, as well as perioperative ultrasounds and liver biopsies. Liver ultrasonography is always necessary to define the diagnosis of liver involvement. Peritoneal metastases are searched, and finally, the ovaries are examined to detect invasion.

### 2.5. Cholecystectomy

Somatostatin analogs represent a main part of the postoperative (in case of R1 and R2 resection) oncological treatment. Those molecules are known to expose patients to the risk of gallbladder stone formation. Prophylactic cholecystectomy can be safely performed without major increase in postoperative morbidity in cholecystectomy vs. no cholecystectomy groups (11.8% vs. 11.1%, respectively; *p* = 0.79) or mortality (1.4% vs. 0.6%, respectively; *p* = 0.29) [20]. To prevent any gallbladder complication (or necrosis in case of future arterial embolization), a prophylactic cholecystectomy should be performed [1]. Therefore, further prospective studies should be performed to identify which patients may benefit from this approach, as stated by Sinnamon et al. [20].

### 2.6. Extensive Lymphadenectomy

To date, five teams have reported positive oncological results arguing for extensive lymph node dissection (Table 1).

Landry et al. described a threshold of seven harvested nodes to have a positive impact on specific survival. The main limitation of this study was that 30% had no lymph nodes analyzed on the surgical specimen [9]. Pasquer et al. reported a low lymph node recurrence rate of 12% with a 5 year recurrence-free survival of 88% in case of extensive curage with a mean follow-up of 54 months. The benefit on overall survival was not achieved due to a lack of statistical power [6]. More recently, Motz et al. described a 79.3% rate of node positivity in 11,852 patients, despite the fact that 46.9% of patients had primary lesions of less than 1 cm. A threshold of eight nodes was used to reliably identify patients with or without lymph node metastasis. Finally, an extensive lymph node resection with a reduced ratio of invaded to benign nodes was predictive of survival [21]. A later study by Zaidi et al. reported that the presence of at least four invaded nodes was significantly associated with poorer 3 year recurrence-free survival (82% vs. 92% if <4N+; *p* = 0.01). In addition, patients with more extensive lymph node resection (≥8 nodes) had different survivals based on the number of nodes involved (3 year recurrence-free survival: 93%, 90%, and 80% if 0, 1–3, ≥4N+, respectively; *p* = 0.047) [23]. Finally, Cives et al. reported on 129 patients with siNETs operated on with oncological data. Regarding lymph node resection, a threshold of more than 17 harvested nodes was associated with the recurrence risk and shorter disease-free survival [22]. 

**Figure 3:**
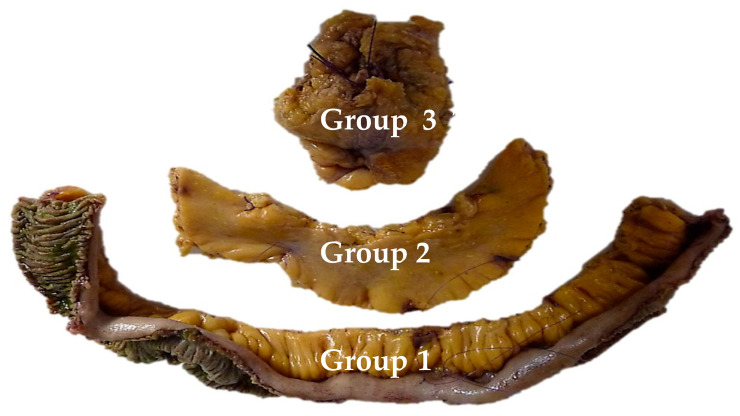
Showing the three nodal groups whose resection is recommended. Figure issued from Pasquer, A., Walter, T., Rousset, P., et al. Lymphadenectomy during Small Bowel Neuroendocrine Tumor Surgery: The Concept of Skip Metastases. *Ann Surg Oncol*. 2016; 23 (Suppl 5): 804–808. Doi: 10.1245/s10434-016-5574-8. Reproduced with permission of Springer nature [23].

The definition of an extensive lymph node dissection is still debated. However, complications related to the mesenteric mass appear when the compression is proximal, either by further progression or by lymph node recurrence. Three lymph node groups have been described: group 1 in contact with the small bowel, group 2 in the middle of the mesentery, and group 3 at the origin of the mesenteric vessels under the pancreatic uncus. In the cohort of Pasquer et al. [24], the phenomenon of skip metastases was objectified (Figure 3). This nodal bypass corresponds to an involvement of the proximal lymph nodes (i.e., groups 2 and 3) while the lymph nodes in contact with the intestine (group 1) are not invaded. This phenomenon appeared in 66% of patients. These data published in 2016 were updated locally in 2019, finding those skip metastases in 69% of cases, but not published.

### 2.7. Resection of the Primitive Lesions and Mesenteric Tumor—Technical Points

**Figure 4:**
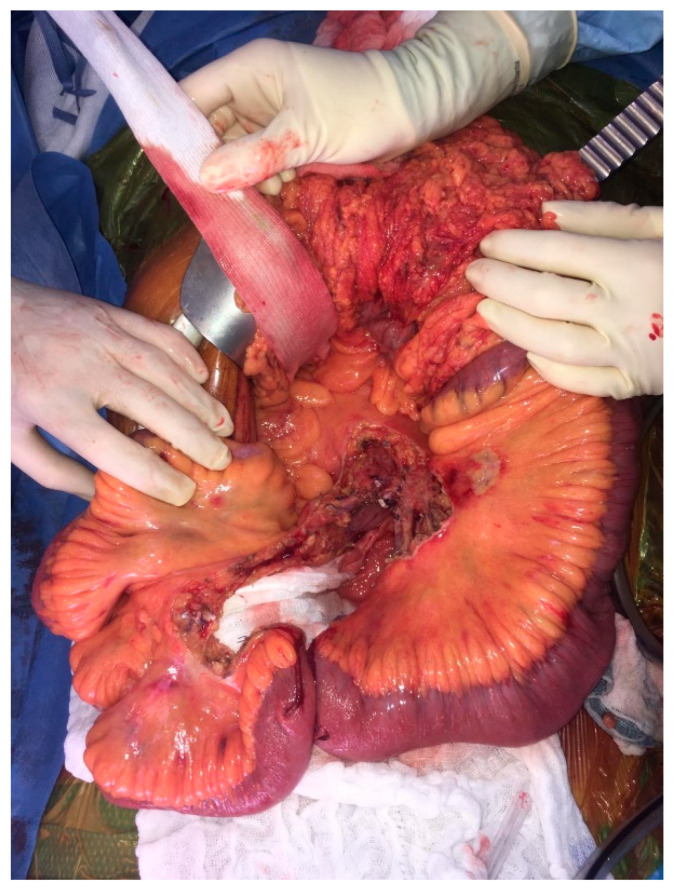
Operative view showing the persisting small bowel after resection (on the proximal side, 2.50 m was preserved; on the distal segment, 1 m was persistent).

**Figure 5:**
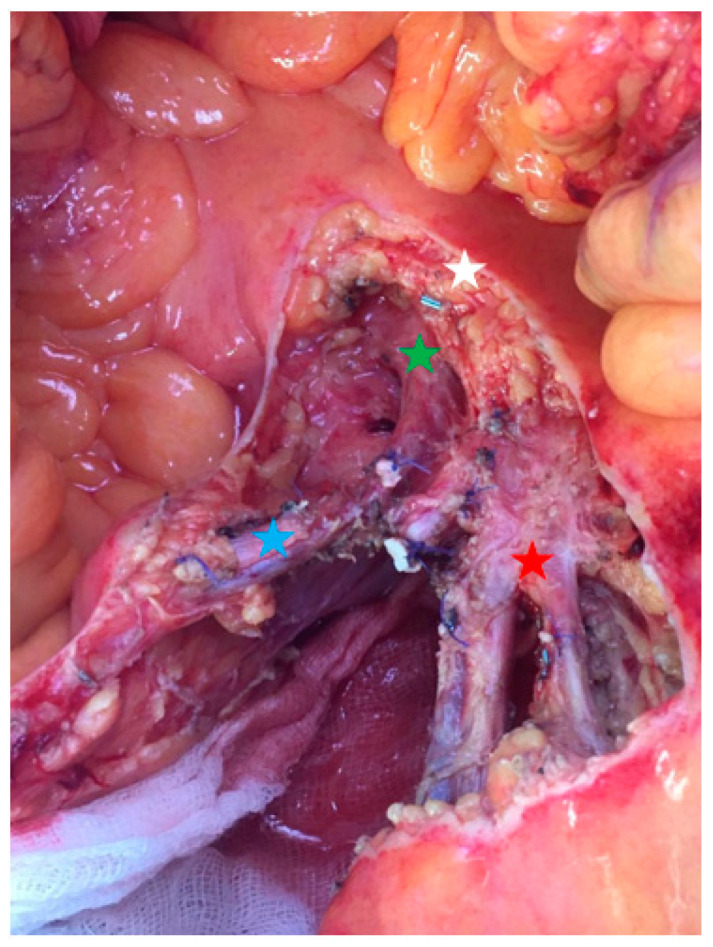
Operative view focusing on the proximal nodal resection (group 3). Red star: proximal jejunal arteries and veins; blue star: superior right colic artery and vein; green star: superior mesenteric vein; white star: pancreas.

The resection starts with dissection of the mesenteric vessels to determine whether the mesenteric nodal block is resectable. Then, the retroperitoneum is opened, followed by a Kocher maneuver to expose the proximal part of the superior mesenteric artery. This strategy allows progressive ligation of vascular branches without any threat to uninvaded jejunal arteries. Lymphadenectomy is performed above the right colic vessels if the nodal mass is located on the right part of the mesenteric axis. If the nodal mass only implies the left side of mesenteric artery, the right superior colic artery can be preserved if the mass is located below its origin. Right hemicolectomy is not mandatory in all patients. Next, the dissection is pursued on the left border of the superior mesenteric vessels. A minimum of three jejunal branches have to be free to avoid the risk of short bowel length [6,13]. Then, the length of the devascularized small bowel determines the resection’s limit (Figure 4). Due to the collateral circulation, the ischemic small bowel segment is usually shorter than expected. Extensive lymphadenectomy can be performed from the small bowel section to the superior mesenteric dissection, with special attention paid to any jejunal pedicle that could be preserved (Figure 5). The length of the remaining small bowel should be measured and recorded. Techniques of reconstruction do not differ from any digestive surgery anastomosis and should be performed according to the experience of the operator.

### 2.8. Operating Report

To date, there are no recommendations concerning the content of the operative report. Nevertheless, we have standardized it and propose the following content.

Concerning the clinical history, it must be complete, associating all the preoperative morphological and biological workup, including the stability of the carcinoid syndrome and the preoperative preparation with octreotide.

Concerning the procedure, the exploration phase must be meticulously described, including:

- At the hepatic level: the presence of a miliary, the location of lesions, the realization of biopsy, and ultrasonography report.
- Mapping of the carcinosis.
- Description of ovarian involvement.
- The number of tumors, their location in relation to the mesenteric axis.
- Classification of the mesenteric mass as resectable type 1 to 4.
- Measurements between the Treitz angle and the first lesion, the resected length and the length downstream to the ileocecal valve.
- The need for a right colectomy.

Concerning anesthetic management, it is important to specify the hemodynamic stability and the presence of flushing during the operation (concomitant with hepatic, ovarian, or other mobilization).

### 2.9. Oophorectomy

A systematic bilateral oophorectomy in case of clinical complaint (such as heaviness or pelvic pain) or carcinoid syndrome and in the presence of ovarian metastases [25] should be performed. We also suggest a systematic oophorectomy if a carcinoid crisis occurs when touching or moving the ovaries during dissection. In young patients, oophorectomy should be realized after extemporaneous pathological study proving NET lesions. Finally, oophorectomy should be discussed in postmenopausal women.

## 3. Emergency Surgery

Occlusive syndrome on retractile mesenteritis or mesenteric ischemia constitutes the main emergencies secondary to a complication of siNETs by local invasion. These patients are sometimes diagnosed at the stage of the complication. Current recommendations are not definitive. However, there is expert agreement that it is not reasonable to initiate resection of the mesenteric mass without comprehensive workup and mapping. It would then seem appropriate to treat the complication if there are signs of severity. It is quite possible to medically treat venous ischemia without necrosis before transferring the patient to an expert center. However, in case of occlusion with signs of severity (ischemia, ascites, septic shock, etc.) or acute arterial ischemia, an intervention to overcome this episode should be proposed. Due to the difficulties of exposition induced by the dilation of the bowel, the risk of missing a tumoral lesion is high, as well as the risk of hemorrhage. Mesenteritis makes dissection difficult, and dilatation of the bowel makes the vascular division even more difficult. Surgery in context of emergency will consist of a resection of the ischemic segments or a simple offloading stoma upstream of the mesenteric mass before transferring the patient to an expert center [8]. It appears dangerous in a complicated situation to approach the origin of the mesenteric vessels with a risk of cataclysmic hemorrhage.

## 4. Conclusions

The surgery of siNETs is in constant evolution. Recent studies plead for resection of primary lesions even in metastatic condition in order to preserve patients from the risk of occlusion or mesenteric ischemia. The challenge lies in the ability to propose a resection without imposing short small bowel syndrome on the patients, which is synonymous with quasi-definitive parenteral nutrition as a complement. The oncological benefits supported in the literature led to recent changes in the recommendations of academic societies. The next steps are the dissemination of reproducible quality criteria as described in this manuscript, as well as the definition of expert centers able to perform these procedures (in parallel with the ENETS expert centers from an oncological point of view).

## Figures and Tables

**Table 1:** Summary of extensive lymphadenectomy literature.

Ref.	Year	First Author	Number of Patients	Threshold of Harvested Nodes	Threshold of Invaded Nodes	Main Result
[9]	2013	Landry	1364	7	-	The number of harvested nodes has positive impact on specific survival
[6]	2015	Pasquer	107	-	-	node recurrence rate 12%, 5 year recurrence-free survival 88%
[21]	2018	Motz	11852	8	-	8 nodes are needed to identify patients with nodal metastasis, the rate of harvested node is predictive of survival
[22]	2018	Cives	129	17	-	Harvested nodes was associated to the recurrence risk and shorter disease-free survival
[23]	2019	Zaidi	199	-	4	>4 invaded nodes is significantly associated with poorer 3 year recurrence-free survival

## Data Availability

No new data were created or analyzed in this study. Data sharing is not applicable to this article.
